# Assessing nonresponse bias at follow-up in a large prospective cohort of relatively young and mobile military service members

**DOI:** 10.1186/1471-2288-10-99

**Published:** 2010-10-21

**Authors:** Alyson J Littman, Edward J Boyko, Isabel G Jacobson, Jaime Horton, Gary D Gackstetter, Besa Smith, Tomoko Hooper, Timothy S Wells, Paul J Amoroso, Tyler C Smith

**Affiliations:** 1 Seattle Epidemiologic Research and Information Center, Department of Veterans Affairs Puget Sound Health Care System, Seattle, WA, USA; 2 Department of Epidemiology, University of Washington, Seattle, WA, USA; 3 Department of Deployment Health Research, Naval Health Research Center, San Diego, CA; 4 Analytic Services, Inc. (ANSER), Arlington, VA, USA; 5 Departments of Preventive Medicine and Biometrics, Uniformed Services University of Health Sciences, Bethesda, MD, USA; 6 Madigan Army Medical Center, Fort Lewis, WA, USA

## Abstract

**Background:**

Nonresponse bias in a longitudinal study could affect the magnitude and direction of measures of association. We identified sociodemographic, behavioral, military, and health-related predictors of response to the first follow-up questionnaire in a large military cohort and assessed the extent to which nonresponse biased measures of association.

**Methods:**

Data are from the baseline and first follow-up survey of the Millennium Cohort Study. Seventy-six thousand, seven hundred and seventy-five eligible individuals completed the baseline survey and were presumed alive at the time of follow-up; of these, 54,960 (71.6%) completed the first follow-up survey. Logistic regression models were used to calculate inverse probability weights using propensity scores.

**Results:**

Characteristics associated with a greater probability of response included female gender, older age, higher education level, officer rank, active-duty status, and a self-reported history of military exposures. Ever smokers, those with a history of chronic alcohol consumption or a major depressive disorder, and those separated from the military at follow-up had a lower probability of response. Nonresponse to the follow-up questionnaire did not result in appreciable bias; bias was greatest in subgroups with small numbers.

**Conclusions:**

These findings suggest that prospective analyses from this cohort are not substantially biased by non-response at the first follow-up assessment.

## Background

Intragroup comparisons over time are a key strength of longitudinal cohort studies; a major threat to the validity of results from such studies is nonresponse to follow-up surveys and/or attrition, which can result in a loss of statistical power and bias. When only a subset of all participants provides follow-up information on exposures and outcomes, the participating subset may not be representative of the original sample. Prior studies have found that follow-up responders tend to differ from nonresponders in their sociodemographic and health characteristics. Since it may be difficult or impossible to determine whether nonresponse is related to the outcome under study, great efforts are usually devoted to maximizing participation and minimizing dropout. Despite such efforts, follow-up survey nonresponse is inevitable and the extent to which such nonresponse might bias study results is a methodological issue of high interest and ongoing concern.

Although not entirely consistent, a number of studies have found that individuals with the following characteristics are more likely to drop out of studies: men (vs. women), not married (vs. married), current smokers, lower socioeconomic status, and poorer health [1-4]. Longitudinal studies of elderly adults have noted that follow-up nonresponders are more likely to be older ([5] and references therein), while studies of younger adults have observed the opposite [2,3]. Nevertheless, few studies have investigated factors predicting nonresponse to follow-up surveys in longitudinal cohorts of younger adult participants. Furthermore, although bias is a major concern, most studies that have used various methods to try to account for nonresponse (e.g., inverse probability weighting and multiple imputation) have not detected substantial bias in estimated measures of association [6-10].

The Millennium Cohort is a 22-year prospective cohort study that began enrollment in 2001 and administered its first follow-up assessment in 2004. The cohort comprises a population of relatively young, highly mobile men and women, often exposed to unique and stressful job circumstances. Moreover, extensive information was collected at baseline on mental, physical, and behavioral health, in addition to sociodemographic, service-related, and occupational characteristics. Previously published Millennium Cohort studies [11-20] have included 1) an investigation of differences in early vs. late responders, 2) a comparison of the cohort to the overall military population, 3) analyses to adjust health outcomes based on the inverse of the sampling and response patterns, 4) evaluation of the early mortality experience among Millennium Cohort participants and invited non-participants, and 5) investigations of health characteristics prior to enrollment. These thorough evaluations of possible biases have demonstrated that Cohort members are generally representative of the US military, that health prior to enrollment did not influence participation, and that Cohort questionnaire data are reliable and internally consistent [11-20]. To complement these previous efforts, the objectives of the current study were to: 1) identify sociodemographic, behavioral, military, and health-related factors associated with response to the follow-up questionnaire, and 2) assess the extent to which failure to account for nonresponse may bias measures of associations between predictors and outcomes under investigation.

## Methods

### The Millennium Cohort

The sampling frame and participant recruitment procedures for the Millennium Cohort have been described in detail elsewhere [18]. Briefly, 256,400 military personnel, representing 11.3 percent of the 2.2 million men and women in active service as of October 1, 2000, were invited to participate in the Millennium Cohort Study between July 2001 and June 2003. Female service members, Reserve and National Guard personnel, and those previously deployed were oversampled. Enrollment was conducted by mail and later by electronic mail invitations. The e-mail invitations presented the option to complete the survey using a Web-based, online questionnaire. A total of 77,047 eligible individuals completed the baseline questionnaire; over half of the respondents did so online. Beginning in June 2004, cohort members were recontacted via e-mail and postal service to complete a follow-up survey. Twenty-nine of the responders to the baseline survey were later determined to be ineligible and 157 individuals died before June 2004. Methods for determining vital status are described in detail elsewhere [12]. Of the 76,861 individuals presumed alive at the time of the administration of the follow-up survey (June 2004-February 2006), 55,046 individuals completed it. After excluding 86 individuals with missing responses for covariates (see Statistical analyses section), 76,775 individuals were included in analyses to calculate the propensity score and 54,960 individuals were included in analyses as responders to the follow-up survey. All enrolled subjects provided informed consent. This study was approved by the Institutional Review Board at the Naval Health Research Center, San Diego (protocol number NHRC.2000.0007).

### Strategies for maximizing response

A modified Dillman method was used to maximize participation at baseline and follow-up, and it included an introductory postcard, survey, and reminder postcard mailings, with repeated survey and reminder postcard mailings for nonresponders [21]. Semiannual e-mails and postcards (sent on Memorial Day and Veterans Day) were used to track participants, sustain interest in continued participation, and verify accuracy of contact information [22]. Participants were sent specially designed messages thanking them for their contribution to military service and to the study and directing them to the study Web site to obtain information on study progress and findings and to update their contact information. In addition, the US Postal Service's "Return Service Requested" was used to obtain forwarding addresses on undeliverable postcards.

### Data collection

Demographic and military data were obtained from the electronic personnel files of the Defense Manpower Data Center and included gender; birth date; race/ethnicity; education; marital status; branch of service; service component; military pay grade; military occupation; deployment experience to Southwest Asia, Bosnia, or Kosovo between 1998 and 2000; deployment experience in support of the wars in Iraq and Afghanistan between 2001 and 2006; and military status at follow-up.

Self-reported data on diagnosed medical conditions, symptoms, psychosocial assessment, occupation(s), use of alcohol and tobacco, as well as military-specific and occupational exposures were obtained from the Millennium Cohort baseline questionnaire, which consisted of more than 450 questions. More information about the survey instrument is available elsewhere [18].

### Statistical analyses

To assess differences in terms of demographic, deployment, occupational, and behavioral characteristics between responders and nonresponders to the follow-up questionnaire, we first conducted descriptive analyses using chi-square tests of association. Next, to calculate propensity scores, we conducted multivariable logistic regression with response to the follow-up questionnaire as the outcome variable. In this case, the propensity score can be thought of as the conditional probability that a person responds given the set of covariates. We assumed that data were missing at random (MAR), meaning that the probability of nonresponse at follow-up depended only on observed data. We used the likelihood ratio test to compare models including a given variable versus absence of the variable in the model (the nested model). Variables with *P *values > 0.05 were removed from the model. We considered for inclusion all variables included in Table 1 as well as the following variables: deployment to Southwest Asia, Bosnia, or Kosovo between 1998 and 2000; binge drinking (drinking ≥5 drinks on a single occasion); survey mode (paper vs. Web); body mass index category (<18.5, 18.5-24.9, 25.0-29.9, ≥30 kg/m^2^); and panic syndrome, other anxiety, eating disorder, hypertension, diabetes, chronic fatigue syndrome, and posttraumatic stress disorder (PTSD), which were all coded as yes/no responses. The final model was comprised of the variables presented in Table 1. To assess the robustness of our model inclusion criterion, we also compared nested models using Akaike's Information Criterion (AIC). The AIC is a calculated index that takes into account both the statistical goodness of fit and the number of parameters that have to be estimated to achieve this particular degree of fit by imposing a penalty for increasing the number of parameters. Lower values of the index indicate the preferred model, that is, the one with the fewest number of parameters that still provides an adequate fit to the data. The same factors were retained in our model whether we determined inclusion based on a *P *value < 0.05 or a lower AIC.

**Table 1:** Characteristics of Millennium Cohort Study participants according to response to follow-up and multivariable regression coefficients predicting response (*N *= 76,775)

Characteristic	Follow-up response status				Multivariable logistic regression model	
	
	Follow-up responder *N *= 54,960		Follow-up nonresponder *N *= 21,815		Beta (standard error)	*P *value
	
	*n*	%	*n*	%		
Intercept					-0.24 (0.07)	0.0006
**Demographic and military characteristics obtained from the Defense Manpower Data Center**						
Gender						
Male	40,311	71.7	15,895	28.3	Ref	<0.0001
Female	14,649	71.2	5,920	28.8	0.36 (0.07)	
Age group (years)						
17-24	7,932	54.7	6,576	45.3	Ref	<0.0001
25-34	19,249	71.3	7,766	28.7	0.56 (0.08)	
35-44	19,600	77.4	5,727	22.6	1.03 (0.09)	
>44	8,179	82.4	1,746	17.6	1.26 (0.12)	
Race/ethnicity						
White, non-Hispanic	38,965	72.9	14,468	27.1	Ref	<0.0001
Black, non-Hispanic	6,721	63.5	3,859	36.5	-0.49 (0.03)	
Asian/Pacific Islander	4,859	80.1	1,207	19.9	-0.02 (0.05)	
Native American	453	66.8	225	33.2	-0.10 (0.14)	
Hispanic	3,202	64.9	1,728	35.1	-0.30 (0.05)	
Other	760	69.9	328	30.1	-0.19 (0.09)	
Education						
Less than high school	2,957	62.9	1,743	37.1	Ref	<0.0001
High school diploma or equivalent	21,053	64.1	11,802	35.9	0.28 (0.07)	
Some college	14,593	74.4	5,011	25.6	0.66 (0.08)	
Bachelor's degree	10,353	81.7	2,312	18.3	0.76 (0.16)	
Postgraduate	6,004	86.4	947	13.6	2.37 (1.07)	
Marital status						
Never married	14,548	63.0	8,541	37.0	Ref	<0.0001
Married	36,484	75.3	11,949	24.7	0.10 (0.02)	
Divorced/widowed/separated	3,928	74.8	1,325	25.2	0.04 (0.04)	
Branch of service						
Army	26,261	72.2	10,118	27.8	Ref	<0.0001
Navy/Coast Guard	9,918	70.0	4,259	30.0	-0.20 (0.03)	
Marines	2,257	57.6	1,659	42.4	-0.43 (0.05)	
Air Force	16,524	74.1	5,782	25.9	-0.24 (0.03)	
Service component						
Reserve/Guard	24,084	73.0	8,928	27.0	Ref	<0.0001
Active duty	30,876	70.6	12,887	29.4	0.44 (0.02)	
Military pay grade						
Enlisted	40,089	67.8	19,046	32.2	Ref	0.0005
Warrant officer	1,168	84.5	214	15.5	0.23 (0.10)	
Commissioned officer	13,703	84.3	2,555	15.7	0.20 (0.07)	
Occupational category						
Combat specialists	11,212	73.0	4,153	27.0	Ref	<0.0001
Electronic equipment repair	5,050	74.6	1,717	25.4	0.21 (0.04)	
Communications/intelligence	3,894	71.9	1,523	28.1	0.16 (0.04)	
Health care	6,144	77.0	1,837	23.0	0.14 (0.04)	
Other technical and allied specialists	1,379	70.0	590	30.0	0.08 (0.06)	
Functional support and administration	11,122	72.4	4,236	27.6	0.11 (0.03)	
Electrical/mechanical equipment repair	7,627	67.2	3,725	32.8	0.05 (0.03)	
Craft workers	1,635	68.6	748	31.4	0.08 (0.05)	
Service and supply	4,680	70.1	1,993	29.9	0.06 (0.04)	
Students, trainees, and other	2,217	63.2	1,293	36.8	0.02 (0.04)	
Deployment experience between 2001 and 2006^a^						
None	40,823	70.8	16,799	29.2	Ref	<0.0001
Deployed	14,137	73.8	5,016	26.2	0.08 (0.02)	
Military status at follow-up						
Enlisted	34,324	71.7	13,560	28.3	Ref	<0.0001
Officer	14,727	84.5	2,706	15.5	0.76 (0.18)	
No longer in military, retired	3,848	63.6	2,203	36.4	-1.51 (1.24)	
No longer in military, other	2,120	38.6	3,373	61.4	-1.03 (0.15)	
**Self-reported military, behavioral, and health characteristics**						
Self-reported military exposures						
No reported exposures	39,197	70.9	16,074	29.1	Ref	0.01
Witnessed a person's death due to war, disaster, or tragic event	12,708	73.2	4,652	26.8	0.05 (0.02)	
Chemical or biological warfare agents	1,427	72.6	538	27.4	0.12 (0.06)	
Both	1,628	74.7	551	25.3	0.09 (0.05)	
Chronic drinking						
No	51,186	72.1	19,811	27.9	Ref	0.0192
Yes	3,774	65.3	2,004	34.7	-0.07 (0.03)	
Smoking status						
Nonsmoker	32,102	74.0	11,287	26.0	Ref	<0.0001
Ever/past smoker	13,594	72.8	5,071	27.2	-0.06 (0.02)	
Current smoker	8,332	64.7	4,538	35.3	-0.22 (0.02)	
Unknown	932	50.4	919	49.6	-0.97 (0.05)	
Self-reported general health						
Excellent	11,248	75.0	3,746	25.0	Ref	<0.0001
Very good	22,233	73.3	8,102	26.7	0.06 (0.02)	
Good	16,222	69.6	7,077	30.4	0.04 (0.03)	
Fair	3,467	65.4	1,837	34.6	0.02 (0.04)	
Poor	320	57.9	233	42.1	-0.14 (0.10)	
Unknown	1,470	64.2	820	35.8	-0.24 (0.05)	
Major depressive disorder						
No	53,431	72.0	20,818	28.0	Ref	0.0010
Yes	1,529	60.5	997	39.5	-0.16 (0.05)	
**Survey response characteristics**						
Early response to baseline questionnaire^b^						
No	37,656	68.9	17,019	31.1	Ref	<0.0001
Yes	17,304	78.3	4,796	21.7	0.46 (0.02)	
**Interaction terms**						
Age group × military status at follow-up interaction^c^						<0.0001
Education × military status at follow-up interaction^c^						<0.0001
Gender × education^c^						0.0012
Age group × education^c^						0.0055
Gender × branch of service^c^						0.0239
Race/ethnicity × branch of service^c^						0.0021

^a^Deployment experience from 2001 to 2006 refers to deployments in support of the wars in Iraq and Afghanistan.

^b^Early response was defined as completing the questionnaire prior to September 1, 2001, which was within 2 months of enrollment invitation on July 1, 2001.

^c^Each interaction generated multiple terms. The number of these terms ranged from 4 (gender × education) to 15 (race/ethnicity × branch of service).

To improve the predictive value of our model, we considered the following first-order interaction terms for inclusion based on a review of the scientific literature and the strength of associations in the multivariable analyses (previous step): gender, age, education, and race/ethnicity, each with the others and with each of the following: marital status, military pay grade, military status at follow-up, service branch, and self-reported health status. We also considered interactions between age and deployment experience and age and self-reported military exposures. We used a two-step process to determine which interaction terms to include in the final model. First, we added interaction terms to the main effects model one at a time. The terms with a *P *value > 0.05 were dropped from further consideration. In the second step, we sequentially added the interaction terms with the smallest *P *value (or smallest AIC in cases of the same *P *value) from the first step. Interaction terms with *P *> 0.05 after inclusion of main effects and the other interaction terms were subsequently removed from the model.

We used the inverse of the propensity score calculated from the multivariable logistic regression model described above to calculate a probability weight for each person, although only responders to the follow-up questionnaire were included in subsequent analyses [23]. Individuals with lower propensities for response were weighted more heavily than those with higher propensities, such that a responder with a propensity equal to 0.2 carried a weight of 5 and a responder with a propensity equal to 0.85 carried a weight of 1.18. The sum of the assigned weights is equal to the baseline population (n = 76,775).

To evaluate the extent to which nonresponse may have influenced measures of association, we used logistic regression along with survey commands that allowed for weighting responders according to their propensity score-derived weights ("weighting for nonresponse"). We selected three outcomes that had been previously studied using Millennium Cohort Study data: disordered eating, depression, and PTSD [24-26]. These outcomes were chosen to include a range of important physical and mental health outcomes. We used the same exclusion criteria and adjusted for the same factors as in the original published studies in order to compare nonresponse-weighted results with the published findings [24-26]. Additionally, to evaluate whether weighting for nonresponse affected estimates of self-reported health at follow-up, we conducted a fourth analysis with self-reported health (five-level variable: excellent, very good, good, fair, poor) as the outcome. For this analysis, we adjusted the proportions based on the propensity score-derived weights.

## Results

Table 1 shows the distribution of demographic and military characteristics ascertained from the DMDC; self-reported military, behavioral, and health characteristics; and survey response characteristics among responders and nonresponders. Overall, 71.6 percent (*n *= 54,960) of individuals completed the first follow-up survey. The response proportion was 10 or more percentage points above average (i.e., ≥81.6 percent) in the following subgroups: age ≥44 years, educational level of a bachelor's degree or higher; and rank of warrant or commissioned officer. The response proportion was 10 or more percentage points below average (≤61.6 percent) among individuals who were aged 17-24 years, no longer in the military at follow-up for reasons other than retirement, Marine Corps service members, missing smoking status, and those who reported having poor health or a major depressive disorder.

Table 1 also includes the adjusted beta coefficients and *P *values for the full propensity score model for each stratum compared with its reference category. A coefficient <0 indicates that the multivariable-adjusted probability of response is smaller than the reference category (equivalent to an odds ratio [OR] <1). Conversely, coefficients >0 indicate that the multivariable adjusted probability of response is larger than the reference category (equivalent to OR >1). To describe an individual's estimated multivariable response probability, beta coefficients across the various characteristics are summed. Since the focus of this analysis was on development of the propensity scores, and not specific ORs, coefficients for each interaction term are not shown in Table 1; the following example is provided for illustration. Women in the Army with less than a high school education (the reference category) were more likely to respond than men in the Army with less than a high school education (OR = exp[0.36] = 1.43). In contrast, women in the Army with a bachelor's degree were no more likely to respond than men in the Army with the same level of education (OR = exp[0.36 +(-0.33)] = 1.03).

**Figure 1:**
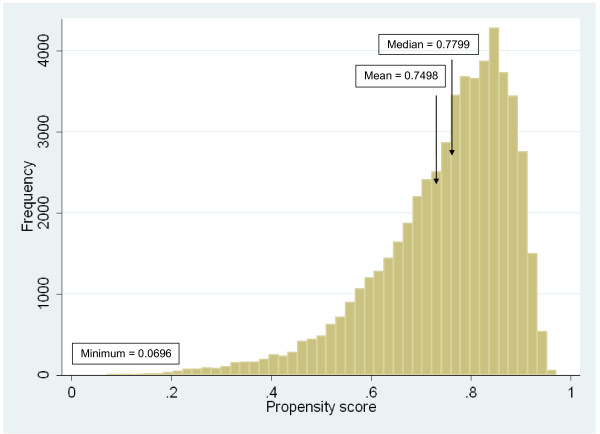
Propensity score statistics (*N *= 54,960 responders)

Figure 1 presents information about the distribution of propensity scores. The mean propensity score was 0.75, indicating that the average weight given to each follow-up responder was 1.33, while the minimum and maximum weights were 1.03 and 14.4, (corresponding to maximum and minimum propensity scores of 0.97 and 0.0696, respectively). The C statistic, a measure of the goodness of fit for the model, was equal to 0.71.

Tables 2, 3, and 4 present ORs and 95 percent confidence intervals (CIs) for the complete case analysis (i.e., "unweighted" results, ignoring nonresponse) and weighted for nonresponse for the association between new-onset eating disorders (Table 2), depression (Table 3), and PTSD (Table 4), respectively, and various exposures, including deployment experience, history of alcohol misuse, and smoking status. In the unweighted analyses, the following characteristics were associated with increased risks of new-onset eating disorders (Table 2): a history of diagnosed mental disorders (men and women), being on a special diet for weight loss (men and women), being active duty (vs. Reserve/Guard, men only), and a history of major life stressors or alcohol misuse (both for men only). After weighting for nonresponse, there was little change in ORs for any strata and no change in the interpretation of results. In some cases, the 95 percent CIs were slightly wider for the nonresponse-weighted estimates. Associations between new-onset depression and deployment experience, smoking status, problem drinking, and PTSD at baseline among men and women were similar with and without weighting for nonresponse (Table 3), although 95 percent CIs were again slightly wider in some cases after weighting for nonresponse.

**Table 2:** Comparison of associations of new-onset eating disorders based on complete case results (ignoring nonresponse) and weighted for nonresponse

	Adjusted odds ratio (95% CI) of new-onset eating disorders			
	
	Women (*N *= 12,641)		Men (*N *= 33,577)	
	
	Complete case analysis	Weighted for nonresponse	Complete case analysis	Weighted for nonresponse
**Deployment experience**				
Nondeployed	1.00	1.00	1.00	1.00
Deployed without combat exposures	0.83 (0.56, 1.23)	0.89 (0.60, 1.32)	0.91 (0.73, 1.13)	0.90 (0.72, 1.13)
Deployed with combat exposures	1.29 (0.91, 1.85)	1.33 (0.93, 1.90)	0.94 (0.77, 1.15)	0.96 (0.77, 1.18)
**Service component**				
Reserve/Guard	1.00	1.00	1.00	1.00
Active duty	1.19 (0.95, 1.48)	1.20 (0.96, 1.50)	1.28 (1.10, 1.49)	1.35 (1.14, 1.60)
**Life stressor scale category**				
Low/mild	1.00	1.00	1.00	1.00
Moderate	1.12 (0.89, 1.43)	1.18 (0.92, 1.52)	1.15 (0.92, 1.44)	1.15 (0.90, 1.46)
Major	1.24 (0.84, 1.82)	1.20 (0.81, 1.77)	1.75 (1.18, 2.57)	1.77 (1.17, 2.68)
**History of diagnosed mental disorder**				
No	1.00	1.00	1.00	1.00
Yes	1.83 (1.45, 2.32)	1.79 (1.40, 2.30)	1.88 (1.51, 2.34)	2.07 (1.61, 2.67)
**History of alcohol misuse**				
No	1.00	1.00	1.00	1.00
Yes	1.29 (0.99, 1.68)	1.27 (0.96, 1.68)	1.44 (1.24, 1.67)	1.52 (1.29, 1.79)
**Special diet for weight loss**				
No	1.00	1.00	1.00	1.00
Yes (Diet)	2.26 (1.84, 2.78)	2.27 (1.82, 2.82)	2.54 (2.15, 2.99)	2.42 (2.04, 2.88)

Abbreviations: CI, confidence interval

Details on exclusion criteria and adjustment factors have been published elsewhere [13].

**Table 3:** Comparison of associations of new-onset depression based on complete case results (ignoring nonresponse) and weighted for nonresponse

	**Adjusted odds ratio **(**95% CI) of new-onset depression**			
	
	Women (*N *= 10,178)		Men (*N *= 30,041)	
	
	Complete case analysis	Weighted for non- response	Complete case analysis	Weighted for non- response
**Deployment experience**				
Nondeployed	1.00	1.00	1.00	1.00
Deployed without combat exposures	0.65 (0.47, 0.89)	0.61 (0.44, 0.84)	0.66 (0.53, 0.83)	0.65 (0.52, 0.83)
Deployed with combat exposures	2.13 (1.70, 2.65)	1.99 (1.58, 2.50)	1.32 (1.13, 1.54)	1.31 (1.12, 1.55)
**Smoking status**				
Never smoker	1.00	1.00	1.00	1.00
Past smoker	1.30 (1.09, 1.55)	1.34 (1.11, 1.62)	1.18 (1.02, 1.36)	1.23 (1.06, 1.43)
Current smoker	1.35 (1.10, 1.66)	1.42 (1.14, 1.78)	1.52 (1.31, 1.77)	1.57 (1.33, 1.85)
**Problem drinking**				
No	1.00	1.00	1.00	1.00
Yes	1.27 (1.03, 1.57)	1.31 (1.04, 1.63)	1.19 (1.04, 1.37)	1.23 (1.06, 1.43)
**Posttraumatic stress at baseline**				
No	1.00	1.00	1.00	1.00
Yes	2.98 (2.07, 4.28)	3.04 (2.09, 4.43)	4.29 (3.34, 5.50)	3.91 (2.98, 5.13)

Abbreviations: CI, confidence interval

Details on exclusion criteria and adjustment factors have been published elsewhere [25].

**Table 4:** Comparison of associations of new-onset posttraumatic stress disorder based on complete case results (ignoring nonresponse) and weighted for nonresponse

	Adjusted odds ratio (95% CI) of new-onset posttraumatic stress disorder							
	
	Army (*N *= 22,958)		Air Force (*N *= 14,609)		Navy and Coast Guard (*N *= 8,655)		Marines (*N *= 2,077)	
	
	Complete case analysis	Weighted for nonresponse	Complete case analysis	Weighted for nonresponse	Complete case analysis	Weighted for nonresponse	Complete case analysis	Weighted for nonresponse
**Deployment experience**								
Nondeployed	1.00	1.00	1.00	1.00	1.00	1.00	1.00	1.00
Deployed without combat exposures	0.87 (0.64, 1.18)	0.63 (0.44, 0.92)	0.56 (0.35, 0.89)	0.44 (0.26, 0.77)	0.60 (0.35, 1.02)	0.48 (0.25, 0.90)	1.42 (0.57, 3.51)	0.79 (0.27, 2.36)
Deployed with combat exposures	3.59 (3.08, 4.17)	3.55 (3.03, 4.16)	3.38 (2.29, 4.98)	3.61 (2.47, 5.28)	2.48 (1.48, 4.14)	2.27 (1.39, 3.71)	2.78 (1.52, 5.07)	2.87 (1.56, 5.30)
**Gender**								
Male	1.00	1.00	1.00	1.00	1.00	1.00	1.00	1.00
Female	1.70 (1.44, 2.00)	1.66 (1.39, 1.97)	2.00 (1.41, 2.83)	2.11 (1.48, 3.01)	1.73 (1.25, 2.38)	1.70 (1.19, 2.43)	1.92 (0.94, 3.94)	1.49 (0.65, 3.39)
**Smoking**								
Never smoker	1.00	1.00	1.00	1.00	1.00	1.00	1.00	1.00
Past smoker	1.21 (1.03, 1.44)	1.22 (1.02, 1.46)	1.05 (0.73, 1.50)	0.85 (0.58, 1.25)	1.33 (0.94, 1.88)	1.42 (0.97, 2.07)	1.55 (0.83, 2.88)	1.68 (0.79, 3.58)
Current smoker	1.69 (1.42, 2.01)	1.76 (1.46, 2.11)	1.40 (0.94, 2.07)	1.24 (0.82, 1.89)	1.59 (1.08, 2.34)	2.01 (1.30, 3.10)	1.84 (0.94, 3.59)	2.07 (0.97, 4.41)
**Problem drinking**								
No	1.00	1.00	1.00	1.00	1.00	1.00	1.00	1.00
Yes	1.47 (1.25, 1.73)	1.47 (1.23, 1.74)	1.69 (1.17, 2.43)	1.78 (1.20, 2.64)	1.69 (1.23, 2.34)	1.74 (1.23, 2.46)	1.73 (1.00, 2.99)	1.60 (0.84, 3.04)
**Military rank**								
Enlisted	2.20 (1.70, 2.86)	2.31 (1.75, 3.05)	2.31 (1.24, 4.30)	2.89 (1.67, 5.00)	2.14 (1.16, 3.94)	1.99 (0.85, 1.68)	1.92 (0.52, 7.13)	1.56 (0.41, 5.93)
Officer	1.00	1.00	1.00	1.00	1.00	1.00	1.00	1.00

Abbreviations: CI, confidence interval

Details on exclusion criteria and adjustment factors have been published elsewhere [26].

As in the published study by Smith et al. [26], ORs for the associations of deployment experience, gender, smoking status, problem drinking, military rank, and new-onset PTSD were stratified by service branch (Army, Air Force, Navy and Coast Guard, and Marines; Table 4). The number of new-onset PTSD cases by service branch was 906 for Army, 184 for Air Force, 195 for Navy and Coast Guard, and 62 for Marine Corps. Particularly for Marines, the sample sizes were small and resulted in relatively imprecise OR estimates. In Army, Navy, and Coast Guard members, deployment without combat exposures was associated with statistically significant reductions in new-onset PTSD in the nonresponse weighted analyses, but not in the complete case analyses (nonresponse weighted analyses: Army, OR: 0.63, 95 percent CI: 0.44, 0.92; Navy/Coast Guard, OR: 0.48, 95 percent CI: 0.25, 0.90). Weighting for nonresponse in analyses of Marines resulted in a shift in ORs from greater than one to less than one, but the 95 percent CIs for both the unweighted and weighted analyses included the null value. For all service branches, the associations between deployment with combat exposure and risk of new-onset PTSD remained strong and positive after weighting. In the unweighted analyses, problem drinking in Marines was associated with a 73 percent increased risk of PTSD, which was of borderline statistical significance (OR = 1.73, 95 percent CI: 1.00, 2.99); after weighting for nonresponse, the point estimate was attenuated toward the null and the confidence limits widened to include 1.0 (OR: 1.60, 95 percent CI: 0.84, 3.04). Similarly, the association between enlisted rank and new-onset PTSD among Navy and Coast Guard members was no longer statistically significant (unweighted OR: 2.14, 95 percent CI: 1.16, 3.94; weighted for nonresponse OR: 1.99, 95 percent CI: 0.85, 1.68). Nevertheless, in both cases, the point estimates did not change substantially.

There was little difference in the distribution of self-reported health at follow-up, with and without weighting for nonresponse (data not shown). Adjusting for nonresponse resulted in a slightly greater proportion of individuals classified as having poor (0.9 percent weighted for nonresponse vs. 0.8 percent unweighted), fair (8.7 percent weighted for nonresponse vs. 8.1 percent unweighted), and good (35.4 percent weighted for nonresponse vs. 34.5 percent unweighted) health, and a smaller proportion classified as reporting very good (38.4 percent weighted for nonresponse vs. 39.2 percent unweighted) or excellent heath (15.3 percent weighted for nonresponse vs. 16.1 percent unweighted).

## Discussion

Using baseline and follow-up data from the Millennium Cohort Study, we evaluated nonresponse bias in a large, relatively young, mobile population of military personnel. A large number of factors were independently associated with response to the follow-up questionnaire. The characteristics associated with a greater probability of response included female gender, increasing age, higher education level, ever married, officer rank, active duty, and self-reported history of military exposures prior to 2001 (vs. none). Ever smokers, those with a history of chronic alcohol consumption or a major depressive disorder at baseline, and those who separated from the military at follow-up, either for retirement or other reason, had a lower probability of response to the follow-up questionnaire. There was no difference in response by history of PTSD, panic disorder, or mode of response (i.e., paper vs. Web) and, thus, these characteristics were not included in Table 1 or subsequent models. Not surprisingly, many of the factors associated with response to the follow-up questionnaire were the same ones associated with response to the baseline questionnaire, namely female gender, older age, non-Hispanic White or Asian/Pacific Islander race/ethnicity, higher education level, ever being married, in the Army or Air Force, warrant or commissioned officer, and from health care or functional support and administration occupations (See Additional File 1) [18]. Many of these characteristics (e.g., female gender, older age, and higher education level) have commonly been associated with greater levels of response [27-29].

The use of propensity scores and weighting for nonresponse allowed us to determine if previous estimates made using complete case analysis were affected by nonresponse. Our results indicated that nonresponse did not substantially affect our estimates of health outcomes related to deployment or other risk factors. Moreover, the self-reported general health of cohort members at follow-up did not appear to be different after weighting for nonresponse. Only where the precision of estimates was low (e.g., new-onset PTSD among Marines) was there a meaningful change in the point estimates that would affect interpretation. Nevertheless, even in this example, since the results from both the unweighted and the weighted analyses were imprecise, it would be imprudent to draw specific conclusions using either method.

There are several limitations that should be considered when interpreting our results. First, since we were unable to collect self-reported follow-up data on nonresponders, we weighted responses/outcomes among responders based on a large number of characteristics to reflect responses of nonresponders at follow-up. Also, we assumed that the data were MAR and if this assumption was invalid, we may not have been successful in adjusting for nonresponse. However, the fact that so much data were collected at baseline reduces the likelihood that some unmeasured factors that are associated with nonresponse were not captured [23]. Second, we were unable to determine whether people did not respond due to refusal (i.e., they received the questionnaire, but chose not to complete it) or inability to be contacted (e.g., the questionnaire was never received due to a change in address, deployment or occupational situation prevented contact via postal or electronic mail, or blocked e-mails). With such a high rate of operational tempo over the past decade, maintaining contact with military personnel who deploy frequently and for sometimes lengthy periods of time or who are required to move and backfill positions around the country has been a challenge. It is plausible that frequent residential moves (typical of the current military lifestyle) may be unrelated to health outcomes. Nevertheless, we were unable to determine whether nonresponse was associated with outcomes under study and potentially incompatible with the MAR assumption. To obtain a better understanding of the possible reasons for nonresponse and characteristics of nonresponders, in 2005, a study of 3,000 nonresponders was conducted (RTI International, unpublished manuscript). Ultimately, 30 percent (*n *= 908) of the nonresponders were contacted and agreed to answer questions. Self-reported health status appeared to differ somewhat between responders to the ancillary study (but nonresponders to the survey in the current study) and responders. Ancillary study responders ("nonresponders") were more likely to report excellent (25 percent vs. 16 percent weighted for survey nonresponse) and fair (10 percent vs. 8 percent weighted for survey nonresponse) or poor (2.1 percent vs. 0.8 percent weighted for survey nonresponse) health compared with responders. However, these data are difficult to interpret since those agreeing to participate in the ancillary study are not likely to be a representative, random sample of all study nonresponders, and instead may represent a subset of individuals who were too busy (reflected in the greater proportion reporting excellent health) or too sick (reflected in the greater proportion reporting poor health) to respond to the initial survey. A third potential limitation was our ability to adequately model response. If response was not adequately modeled, our ability to adjust for nonresponse would be diminished. However, this seems unlikely since to create the propensity score, we evaluated a large number of characteristics among baseline responders, including demographic, military, behavioral, and medical characteristics and the C statistic of 0.71 indicated a good fit of the model.

## Conclusions

In summary, we found that in this relatively young adult, highly mobile cohort, several factors previously identified (e.g., male gender, younger age, lower education), as well as some novel factors (e.g., separation from the military), were associated with lower probability of response. To reduce nonresponse in future follow-up surveys, it will be important to put additional efforts into maintaining contact and encouraging participation for individuals with these characteristics. Furthermore, because individuals who separate from the military (or analogously for an occupational cohort, individuals who are no longer working in the industry) may incorrectly assume that they are no longer enrolled in the study, it will be helpful to continue to employ strategies for the future follow-up cycles that proactively inform these individuals regarding the importance of their continued participation, regardless of their current occupational status. In this study population, nonresponse to the follow-up questionnaire did not result in appreciable bias as reflected by comparing measures of association for selected outcomes using complete case and inverse probability weighted methods. The potential for bias seemed greatest in subsamples with smaller numbers, as there were slight differences in point estimates and precision obtained from these two methods of analysis. Nevertheless, there is no substitute for adequate follow-up to support proper epidemiologic inference; efforts to achieve and maintain high response rates are a worthwhile investment in this, and all prospective cohort studies.

## Abbreviations

AIC: Akaike's Information Criterion; CI: confidence interval; MAR, missing at random; OR: odds ratio; PTSD: posttraumatic stress disorder

## Competing interests

The authors declare that they have no competing interests.

## Authors' contributions

AJL advised on the approach for statistical analyses and drafted the manuscript. EJB conceived the idea for the study and advised on the approach for statistical analyses. JH and IGJ performed the statistical analyses. All authors participated in the design of the study, edited and revised several drafts, and read and approved the final manuscript.

## Pre-publication history

The pre-publication history for this paper can be accessed here:

http://www.biomedcentral.com/1471-2288/10/99/prepub

## Supplementary Material

Additional file 1 Distribution of various demographic and military characteristics in the sample invited to participate in the Millennium Cohort Study in 2001 and responders to the first follow-up survey in 2004. Click here for file
