# Supplementary material for: Assessing nonresponse bias at follow-up in a large prospective cohort of relatively young and mobile military service members
Source: BMC Med Res Methodol. 2010 Oct 21;10:99. doi: 10.1186/1471-2288-10-99 (PMC2984503; doi:10.1186/1471-2288-10-99)
Supplement: Additional file 1 — Distribution of various demographic and military characteristics in the sample invited to participate in the Millennium Cohort Study in 2001 and responders to the first follow-up survey in 2004. [file 1471-2288-10-99-S1.DOC]

Additional File 1. Distribution of various demographic and military characteristics in the sample invited to participate in the Millennium Cohort Study in 2001 and responders to the first follow-up survey in 2004.

| **Demographic and military characteristics** | Invited sample  (*N*=256,400) | Responders to 1^st^ follow-up survey  (*N*=54,960) |
| --- | --- | --- |
|  | % | |
| Gender |  |  |
| Male | 76.0 | 73.3 |
| Female | 24.0 | 26.7 |
| Age group (years) |  |  |
| 17-24 | 30.8 | 14.4 |
| 25-34 | 35.4 | 35.0 |
| 35-44 | 25.1 | 35.7 |
| >44 | 8.6 | 14.9 |
| Race/ethnicity |  |  |
| White, non-Hispanic | 64.7 | 70.9 |
| Black, non-Hispanic | 19.0 | 12.2 |
| Asian/Pacific Islander | 6.1 | 8.8 |
| Native American | 0.9 | 0.8 |
| Hispanic | 7.5 | 5.8 |
| Other | 1.5 | 1.4 |
| Education |  |  |
| Less than high school | 7.6 | 5.4 |
| High school diploma or equivalent | 50.4 | 38.3 |
| Some college | 23.6 | 26.6 |
| Bachelor’s degree | 11.6 | 18.8 |
| Postgraduate | 5.4 | 10.9 |
| Marital status |  |  |
| Never married | 40.5 | 26.5 |
| Married | 52.8 | 66.4 |
| Divorced/widowed/separated | 5.7 | 7.1 |
| Branch of service |  |  |
| Army | 44.0 | 47.8 |
| Navy/Coast Guard | 20.8 | 18.0 |
| Marines | 7.2 | 4.1 |
| Air Force | 28.1 | 30.1 |
| Service component |  |  |
| Reserve/Guard | 45.1 | 43.8 |
| Active duty | 54.9 | 56.2 |
| Military pay grade |  |  |
| Enlisted | 84.6 | 72.9 |
| Warrant officer | 1.1 | 2.1 |
| Commissioned officer | 14.3 | 24.9 |
| Occupational category |  |  |
| Combat specialists | 20.9 | 20.4 |
| Electronic equipment repair | 8.0 | 9.2 |
| Communications/intelligence | 6.7 | 7.1 |
| Health care | 8.4 | 11.2 |
| Other technical and allied specialists | 2.4 | 2.5 |
| Functional support and administration | 17.9 | 20.2 |
| Electrical/mechanical equipment repair | 16.2 | 13.9 |
| Craft workers | 3.5 | 3.0 |
| Service and supply | 8.9 | 8.5 |
| Students, trainees, and other | 5.8 | 4.0 |
